# Biomarkers to Predict Acute Kidney Injury in Patients with Trauma

**DOI:** 10.3390/medicina61101853

**Published:** 2025-10-16

**Authors:** In Sik Shin, Myoung Jun Kim, Da Kyung Kim, Joon Hyeong Sohn, Kwangmin Kim

**Affiliations:** 1 Division of Acute Care Surgery, Wonju College of Medicine, Yonsei University, Wonju 26426, Republic of Korea; 2 Trauma Center, Armed Forces Capital Hospital, Sungnam 13574, Republic of Korea; 3 Hyperbaric Oxygen Therapy Research Institute, Wonju College of Medicine, Yonsei University, Wonju 26426, Republic of Korea; 4 Central Research Laboratory, Wonju College of Medicine, Yonsei University, Wonju 26426, Republic of Korea; 5 Health Check-Up Center, Wonju Severance Christian Hospital, Wonju 26426, Republic of Korea; 6 Center of Evidence Based Medicine, Institute of Convergence Science, Yonsei University, Seoul 03722, Republic of Korea; 7 Institute of Evidence Based Medicine, Wonju College of Medicine, Yonsei University, Wonju 26426, Republic of Korea

**Keywords:** trauma, acute kidney injury, biomarkers, mitochondria, haemoglobin, mtDNA

## Abstract

*Background and Objectives*: Acute kidney injury (AKI) is a common complication in patients with trauma and is associated with increased morbidity and mortality rates. Early identification of patients at risk of AKI may enable timely intervention and improved outcomes. Biomarkers such as urinary mitochondrial DNA copy number (mtDNAcn) may play a role in predicting AKI. However, its role as a predictor of AKI has rarely been studied in patients with trauma. Therefore, the aim of this study was to evaluate the utility of mtDNA for early detection of AKI in this patient population. *Materials and Methods*: This single-center prospective observational study included patients with trauma admitted to a regional trauma center between July 2022 and July 2023. Serum and urine samples were collected at baseline and at 24, 48, and 72 h to measure mtDNAcn using real-time polymerase chain reaction test. Clinical variables, including hemoglobin (Hb) levels, were also recorded. *Results*: Among 65 enrolled patients, 25 (38.5%) developed AKI. Patients with AKI showed significantly lower Hb levels and higher urinary mtDNAcn at admission. Multivariate logistic regression analysis identified low Hb and elevated urinary mtDNAcn as independent predictors of AKI. The optimal cutoff value was 10.95 g/dL for Hb and 738.0 copies/μL for urinary mtDNAcn. However, no significant temporal differences in serum mtDNAcn were observed between the AKI and no-AKI groups. *Conclusions*: Both Hb and urinary mtDNAcn may serve as independent biomarkers for early identification of AKI in patients with trauma. Future studies are warranted to determine optimal targets and validate these findings in larger multicenter cohorts.

## 1. Introduction

Patients with trauma are vulnerable to acute kidney injury (AKI) [1]. Several factors contribute to AKI development in this population, including renal hypoperfusion due to hypovolemic shock, rhabdomyolysis, abdominal vascular compression due to increased intra-abdominal pressure, and nephrotoxic drugs used during treatment [1,2].

AKI in patients with trauma is of considerable clinical significance. It can act as a precursor to multi-organ dysfunction syndrome (MODS) in these patients and is strongly correlated with adverse outcomes, including increased mortality rates [2].

Early detection of AKI in critically ill patients is important, as it plays a pivotal role in optimizing therapeutic interventions and mitigating the risk of associated complications. Therefore, research has been focused on evaluating various molecular biomarkers to improve early diagnosis and prognosis of AKI in critically ill patients [2].

Among these biomarkers, we focused on mitochondrial DNA (mtDNA), given the crucial role of mitochondrial integrity in the development of AKI. Early in the onset of AKI, structural and functional alterations occur in the renal tubular epithelium, including reduced mitochondrial abundance, organelle swelling, and mitochondrial fragmentation [3]. This mitochondrial deficiency leads to kidney injury through the generation of reactive oxygen species (ROS) and release of mtDNA [4]. The secreted mtDNA binds to toll-like receptor 9 (TLR9), thereby activating innate immune responses [4]. Additionally, mtDNA acts as a damage-associated molecular pattern (DAMP), triggering inflammatory responses that ultimately contribute to organ damage [5,6].

Although some studies have investigated the association between mtDNA and AKI in critically ill or surgical patients [7,8], the role of mtDNA as a predictor of AKI has rarely been studied in patients with trauma. Given its involvement in molecular tissue damage from the early stages of AKI, it is expected to be a promising marker for early detection of AKI.

Among various molecular biomarkers such as NGAL (neutrophil gelatinase-associated lipocalin), KIM-1 (kidney injury molecule-1), and β2-microglobulin, we focused on mtDNA, given the crucial role of mitochondrial integrity in the development of AKI. Therefore, the aim of this study was to evaluate the utility of mtDNA for the early detection of AKI in trauma patients.

## 2. Materials and Methods

### 2.1. Participant Selection

This prospective observational study, incorporating experimental assays to evaluate serum and urine mtDNA as predictors of AKI, was conducted at a single regional trauma center in South Korea. This study was approved by the Institutional Review Board of Wonju Severance Christian Hospital (IRB No. CR322047), adhering to the principles outlined in the Declaration of Helsinki. The study protocol was registered at ClinicalTrials.gov (Identifier: NCT05441787). Patients with trauma who presented to our trauma center between July 2022 and July 2023 were enrolled. Patients aged less than 18 years, pregnant individuals, and those presenting with immediate life-threatening conditions were excluded.

### 2.2. Data Collection and Definitions

After enrollment of eligible patients, clinical data were prospectively collected through direct patient history taking at admission and review of the hospital’s electronic medical record system. The following information was recorded: patient age, sex, relevant medical history, mechanism of trauma, vital signs, Abbreviated Injury Scale (score range: 1–6), Injury Severity Score, procedures performed, and initial laboratory findings. Laboratory assessments included the delta neutrophil index, white blood cell count, neutrophil count, creatinine, hemoglobin, platelet count, international normalized ratio, C-reactive protein level, and lactate concentration. Additionally, measurements of serum mitochondrial DNA copy number (smtDNAcn) and urinary mitochondrial DNA copy number (umtDNAcn) were obtained. Blood samples for mtDNA quantification were collected upon arrival and at 24, 48, and 72 h after initial sampling. Sequential Organ Failure Assessment (SOFA) scores were routinely documented throughout the intensive care unit (ICU) admission. Posthospitalization data included number of packed red blood cell (RBC) transfusions administered, total length of hospital stay, duration of ICU stay, and survival status. AKI was diagnosed according to the Kidney Disease: Improving Global Outcomes (KDIGO) criteria.

### 2.3. Procedures for Sampling, DNA Extraction, and Quantification

At admission, venous blood was collected into EDTA-coated tubes, with additional samples obtained at 24, 48, and 72 h. All specimens were delivered to the central laboratory within two hours of collection. Plasma was separated by sequential centrifugation (initially at 1300× *g* for 10 min at 4 °C, followed by a second spin at 4000× *g* for 10 min), after which aliquots of the supernatant were stored at –80 °C. Urine specimens were obtained at the same time points, centrifuged under identical conditions, and supernatants were frozen at −80 °C until analysis. To minimize DNA degradation, repeated freeze–thaw cycles were avoided, and samples were stored for no longer than three months. Cell-free DNA from plasma and urine was isolated using the QIAamp DNA Mini Kit (#51306; Qiagen, Hilden, Germany) in accordance with the manufacturer’s protocol. Briefly, specimens were lysed with buffer and proteinase K, mixed with ethanol, and applied to spin columns. After two washing steps, DNA was eluted in 50 μL of nuclease-free water. Purified DNA (extracted from 200 µL of plasma or urine) was subjected to quantitative PCR using SYBR Green chemistry (QuantStudio 6 Flex Real-Time PCR System, Applied Biosystems, Waltham, MA, USA). Primers targeting human mitochondrial DNA (forward: 5′-CACTTTCCACACAGACATCA-3′; reverse: 5′-TGGTTAGGCTGGTGTTAGGG-3′) were used. A standard curve was generated using serial dilutions of a PCR product (10^8^ to 10^2^ copies/μL). Amplification conditions included an initial denaturation at 95 °C for 10 min, followed by 40 cycles of 95 °C for 20 s and 60 °C for 10 s. Each reaction was performed in triplicate, and negative controls were included. Copy numbers were expressed as copies/μL of plasma or urine, calculated from standard curves and adjusted for sample and elution volumes. More details on blood and urine sampling, sample preparation, storage procedures, DNA extraction, quantitative polymerase chain reaction methods, and the calculation formula for mtDNAcn are provided in our previous study [7].

### 2.4. Statistical Analysis

Statistical analyses were conducted using R statistical software (version 4.4.0; R Foundation for Statistical Computing, Vienna, Austria). Continuous variables were expressed as means with standard deviations. Independent sample t-tests were used to compare normally distributed continuous variables, while the Mann–Whitney U test was applied to variables with non-normal distributions. Categorical variables were compared using the chi-square test or Fisher’s exact test. Multivariate logistic regression analyses were performed to identify independent risk factors. Variables were selected for multivariate logistic regression based on clinical relevance and statistical significance in univariate analyses. Multicollinearity was assessed using variance inflation factors (VIF), all of which were below 3, indicating no significant multicollinearity. Receiver operating characteristic (ROC) curves were generated, and optimal cutoff values for mortality prediction were determined using the Youden index. Statistical significance was set at *p <* 0.05.

## 3. Results

### 3.1. Comparison Between Patients with and Without Acute Kidney Injury

Sixty-five patients with trauma were included, 25 (38.5%) of whom developed AKI. Patients with AKI were significantly older than those without AKI (64.7 ± 14.8 vs. 54.4 ± 18.0 years, *p* = 0.020) and had lower systolic blood pressure values upon admission (98.6 ± 34.8 vs. 117.5 ± 32.3 mmHg, *p* = 0.030) as well as higher injury severity scores (20.0 ± 8.7 vs. 14.9 ± 9.1, *p* = 0.030). Among the enrolled patients, 78 (97.5%) sustained blunt trauma, whereas only 2 (2.5%) had penetrating injuries. Both patients with penetrating trauma were included in the no AKI group, and no patient with AKI had penetrating trauma. Initial laboratory findings revealed higher serum creatinine (1.3 ± 0.4 vs. 0.9 ± 0.3 mg/dL, *p <* 0.001) and lower hemoglobin (11.4 ± 2.3 vs. 12.8 ± 1.8 g/dL, *p* = 0.008) levels in the AKI group. Worst SOFA scores were significantly higher in patients with AKI (5.0 ± 3.0 vs. 2.2 ± 2.3, *p <* 0.001). The number of RBC transfusions within 24 h was higher in the AKI group (4.3 ± 5.3 vs. 1.8 ± 4.0 units, *p* = 0.037), although no significant difference in hospital mortality rate was observed (4.0% vs. 0%, *p* = 0.811) (Table 1).

### 3.2. Temporal Changes in Mitochondrial DNA Copy Number

**Figure 1:**
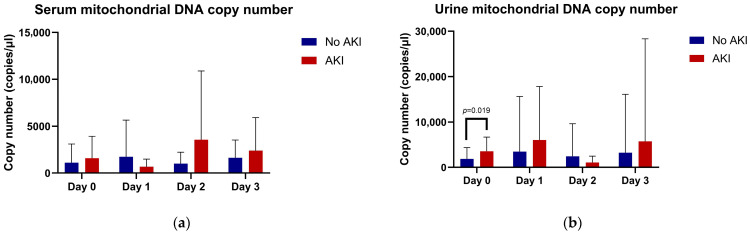
Temporal changes in mitochondrial DNA copy numbers in patients with and without acute kidney injury. (**a**) Serum mitochondrial DNA copy number (smtDNAcn) trends over four days. (**b**) Urine mitochondrial DNA copy number (umtDNAcn) trends over four days.

In the no-AKI group, smtDNAcn increased on day 1, decreased on day 2, and increased again on day 3. In contrast, the AKI group showed a decrease in smtDNAcn on day 1, a marked increase on day 2, and a subsequent decline on day 3. No statistically significant differences in smtDNAcn were observed between the two groups at any of the measured time points from day 0 to day 3 (Table 2, Figure 1a).

For umtDNAcn, the no-AKI group showed a progressive increase from day 0 to day 1, followed by a decline on day 2, and a slight rise again on day 3. In the AKI group, umtDNAcn were significantly elevated on day 0, continued to increase on day 1, and then decreased on day 2 before increasing again on day 3. Notably, a significant difference in umtDNAcn between the two groups was observed on day 0 (*p* = 0.019); however, no significant differences were found at subsequent time points (Table 2, Figure 1b).

### 3.3. Multivariate Analysis for Predicting Acute Kidney Injury

Multivariate logistic regression analysis identified decreased hemoglobin levels (odds ratio [OR], 0.70553; 95% confidence interval [CI], 0.53281–0.93424; *p* = 0.014) and increased initial umtDNAcn (OR, 1.00022; 95% CI, 1.00002–1.00042; *p* = 0.033) as independent predictors of AKI (Table 3).

### 3.4. Optimal Cutoff Values for Hemoglobin and umtDNAcn

**Figure 2:**
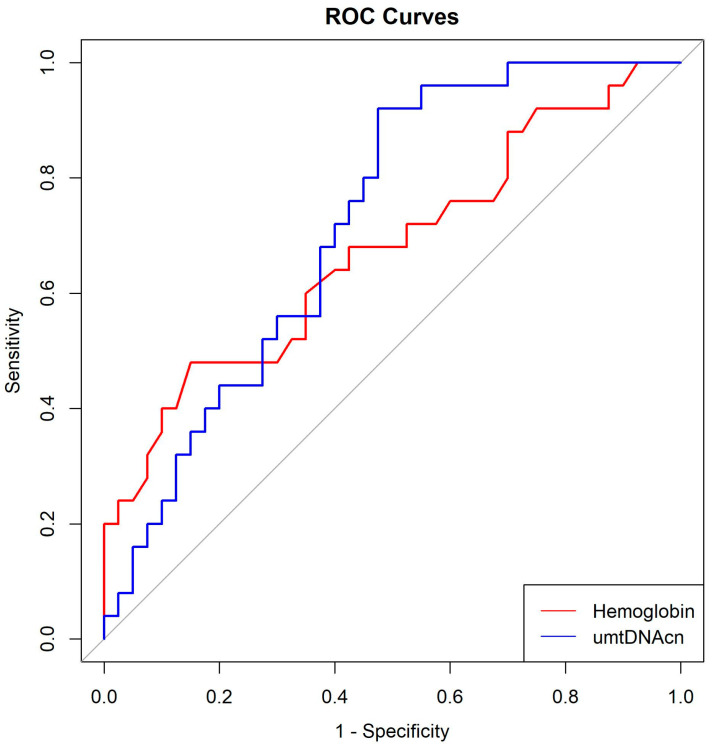
Receiver operating characteristics curves for the hemoglobin, and initial urine mitochondrial DNA copy number between the patients with and without. AKI. umtDNAcn urine mitochondrial DNA copy umber.

ROC curve analysis revealed that the optimal cut-off for hemoglobin to predict AKI was 10.95 g/dL (AUC: 0.6735; sensitivity: 48%; specificity: 85%), and for initial umtDNAcn was 738.0 copies/μL (AUC: 0.7200; sensitivity: 92%; specificity: 52.5%) (Table 4, Figure 2).

## 4. Discussion

This study revealed that umtDNAcn and hemoglobin level were independently associated with AKI in patients with trauma. The optimal cut off value for umtDNAcn was 738.0 copies/μL, and that for initial hemoglobin was 10.95 g/dL.

Mitochondria are key contributor to the development of AKI owing to their dual roles in energy production and regulation of cell death mechanisms. Early mitochondrial alterations have been identified in AKI, particularly in ischemia-related cases, resulting in both decreased mitochondrial quantity and distinct morphological disruptions, such as mitochondrial swelling and loss of cristae in the inner membrane. These structural changes are largely driven by reduced ATP production and the collapse of mitochondrial membrane potential [9]. A critical downstream effect is the opening of mitochondrial permeability transition pores, which facilitate the release of pro-apoptotic molecules, such as cytochrome c, subsequently triggering apoptosis in kidney cells [10].

Disruption of mitochondrial dynamics has also been linked to AKI progression. Specifically, increased mitochondrial fission, mediated by rapid activation of Dynamin-related protein 1 (Drp1), and diminished expression of fusion proteins such as Mitofusin and Optic Atrophy 1 (OPA1), lead to mitochondrial fragmentation and further dysfunction [11].

Mitochondrial injury leads to the extracellular release of mtDNA, which acts as a DAMP. Released mtDNA can activate cytosolic DNA sensors such as cGAS, initiating inflammatory signaling cascades [5,6]. In parallel, mitochondrial ROS contributes to oxidative stress and renal cellular damage [12]. Thus, the release of mtDNA into the urine may reflect mitochondrial dysfunction associated with AKI.

To date, no studies have been conducted to directly investigate umtDNA as a predictive biomarker of AKI in patients with trauma. While elevated umtDNA levels have been associated with the development of AKI in surgical ICU populations [8], such findings have yet to be validated in trauma-specific cohorts. Moreover, a study [13] showed no significant differences in umtDNA levels between patients with and without AKI following cardiac surgery. These inconsistent results, combined with the lack of trauma-focused research, underscore the need for further studies to determine the clinical relevance of urinary mtDNA in predicting AKI in patients with trauma.

In the present study, hemoglobin level was identified as an independent predictor of AKI in trauma patients. This finding highlights the important relationship between anemia and the development of renal dysfunction in the context of acute injury. While trauma-induced AKI is multifactorial, involving hemodynamic instability, rhabdomyolysis, nephrotoxic exposure, and systemic inflammation, our results emphasize the critical role of hemoglobin as a modifiable physiological parameter. Low hemoglobin levels in patients with trauma may reflect acute blood loss, dilutional effects from aggressive fluid resuscitation, or preexisting anemia. In an acute setting, reduced hemoglobin levels compromise oxygen delivery to renal tissue, which is particularly vulnerable to hypoxia owing to its high metabolic demand and limited capacity for oxygen extraction. In particular, the renal medulla is highly susceptible to ischemic injury. Therefore, decreased hemoglobin levels may exacerbate renal hypoperfusion, increasing the risk of ischemia-induced tubular injury and subsequent AKI [1,14]. This mechanism is supported by experimental and clinical data suggesting that anemia aggravates renal hypoxia and enhances susceptibility to nephrotoxic insults [15,16]. Furthermore, Provenzano et al. demonstrated that low hemoglobin levels were associated with an increased renal resistive index in patients with chronic kidney disease [17], suggesting that anemia may also impair intrarenal vascular hemodynamics. This raises the possibility that reduced hemoglobin could contribute to AKI not only through systemic hypoxia but also via altered renal microcirculation and vascular resistance.

Several prior studies have shown findings consistent with our study, identifying low hemoglobin as a risk factor for AKI in various critically ill populations [18,19]. However, other investigations have failed to show a clear association [20,21], possibly because of differences in patient populations, timing of hemoglobin measurement, or thresholds used to define anemia. These conflicting results highlight the complexity of the relationship between anemia and kidney injury. Moreover, anemia may also simply reflect trauma severity, bleeding, or resuscitation practices rather than directly contributing to AKI development; therefore, our findings regarding hemoglobin should be interpreted with caution. Nevertheless, given that hemoglobin is a modifiable factor, further research should be focused on elucidating its role and determining appropriate hemoglobin targets for preventing AKI in patients with trauma.

The incidence of AKI in patients with severe trauma has been reported to range from 6% to 74% [22,23]. This wide variation in incidence across studies can be explained by the use of different diagnostic criteria for AKI, [22] including the Risk, Injury, Failure, Loss of Function, End-stage Renal Disease (RIFLE) criteria [24], Acute Kidney Injury Network (AKIN) criteria [25], and KDIGO criteria [26]. Among these, the KDIGO criteria have the highest sensitivity for diagnosing AKI and are the most effective in predicting mortality [27,28]. In the present study, the incidence of AKI was 38.5%, classified according to the KDIGO guidelines. Given that the KDIGO criteria provide the highest sensitivity and best prognostic value for AKI, the use of this standardized definition ensures that the incidence reported here is reliable and that subsequent risk factor analyses are appropriately based on a robust classification system.

This study has several limitations. First, although we attempted to control for a broad range of variables in our multivariate analysis, unmeasured confounders may have influenced the results. Second, despite efforts to standardize the processing of biological samples, such as initiating preparation within two hours of collection, minor variations in the timing of sample collection and handling may have introduced bias, particularly in the measurement of mtDNAcn. Third, the relatively small sample size, particularly the limited number of patients who developed AKI (n = 25), may have reduced the statistical power and generalizability of our findings.

## 5. Conclusions

Despite these limitations, our findings provide significant insights. To our knowledge, this is one of the few prospective observational studies conducted to systematically investigate and compare multiple biomarkers for AKI, specifically in patients with trauma. In this study, we found that higher urinary mtDNA copy number (umtDNAcn) and lower hemoglobin levels at admission were independently associated with the development of AKI. The optimal cutoff values identified were 738.0 copies/μL for umtDNAcn and 10.95 g/dL for hemoglobin. These findings suggest that both biomarkers may have potential clinical value in the early identification of trauma patients at risk for AKI. Future large-scale, multicenter prospective studies are warranted to validate and extend these results.

## Figures and Tables

**Table 1:** Background characteristics of patients with and without acute kidney injury.

	No AKI (*n* = 40) (%)	AKI (*n* = 25) (%)	*p*-Value
Age (years)	54.4 ± 18.0	64.7 ± 14.8	0.020
Male sex	32 (80.0)	20 (80.0)	1.000
Known history			
Hypertension	12 (30.0)	10 (40.0)	0.576
Diabetes mellitus	5 (12.5)	4 (16.0)	0.977
Cerebrovascular disorder	1 (2.5)	0 (0.0)	1.000
Liver disease	1 (2.5)	1 (4.0)	1.000
Respiratory disease	2 (5.0)	0 (0.0)	0.691
Penetrating injury	2 (5.0)	0 (0.0)	0.691
SBP (mmHg)	117.5 ± 32.3	98.6 ± 34.8	0.030
ISS	14.9 ± 9.1	20.0 ± 8.7	0.030
AIS1	0.6 ± 1.3	0.9 ± 1.2	0.382
AIS2	0.3 ± 0.6	0.5 ± 0.9	0.236
AIS3	1.6 ± 1.5	2.3 ± 1.5	0.067
AIS4	1.7 ± 1.3	1.9 ± 1.2	0.635
AIS5	1.0 ± 1.5	1.2 ± 1.7	0.591
AIS6	0.6 ± 0.6	0.6 ± 0.7	0.678
Initial laboratory findings			
DNI (%)	2.1 ± 2.2	3.0 ± 3.2	0.201
WBC (X^3^) (10^9^/L)	14.4 ± 4.7	15.0 ± 9.2	0.757
Neutrophil (X^3^) (10^9^/L)	11.9 ± 4.2	12.4 ± 8.8	0.801
Creatinine (mg/dL)	0.9 ± 0.3	1.3 ± 0.4	<0.001
Hemoglobin (g/dL)	12.8 ± 1.8	11.4 ± 2.3	0.008
Platelet (X^3^) (10^9^/L)	234.1 ± 111.5	192.6 ± 64.1	0.062
INR	1.1 ± 0.2	1.2 ± 0.1	0.140
CRP (mg/dL)	1.1 ± 2.2	1.9 ± 5.9	0.477
Lactate (mmol/L)	3.0 ± 2.4	4.4 ± 3.2	0.054
Procedure			0.148
Observation only	22 (55.0)	19 (76.0)	
Angioembolization	3 (7.5)	0 (0.0)	
Surgery	15 (37.5)	6 (24.0)	
Worst SOFA score	2.2 ± 2.3	5.0 ± 3.0	<0.001
RBC transfusion ≤ 24 h	1.8 ± 4.0	4.3 ± 5.3	0.037
Hospital LOS	19.9 ± 21.1	28.7 ± 22.5	0.118
ICU LOS	6.6 ± 11.0	7.8 ± 6.1	0.590
Mortality	0 (0.0%)	1 (4.0%)	0.811

SBP, systolic blood pressure; ISS, injury severity score; AIS, abbreviated injury scale; DNI, delta neutrophil index; WBC, white blood cell; INR, international normalized ratio; CRP, C-reactive protein; SOFA, sequential organ failure assessment; ICU, intensive care unit; LOS, length of stay.

**Table 2:** Differences in daily change in serum and urine mtDNAcn (copies/μL) between patients with and without acute kidney injury.

		Day 0	Day 1	Day 2	Day 3
SmtDNAcn (copies/μL)	No AKI	1114.7 ± 1996.2	1749.3 ± 3902.5	1024.7 ± 1207.5	1644.9 ± 1886.8
	AKI	1594.5 ± 2341.6	677.3 ± 811.3	3567.3 ± 7328.4	2401.7 ± 3524.4
	*p*-value	0.381	0.154	0.140	0.387
UmtDNAcn (copies/μL)	No AKI	1896.8 ± 2476.8	3482.4 ± 12,126.2	2472.2 ± 7167.9	3265.6 ± 12,817.8
	AKI	3574.5 ± 3096.5	6040.7 ± 11,803.0	1106.4 ± 1371.5	5747.6 ± 22,591.1
	*p*-value	0.019	0.428	0.287	0.643

smtDNAcn, serum mitochondrial DNA copy number; AKI, acute kidney injury; umtDNAcn, urine mitochondrial DNA copy number.

**Table 3:** Multivariate logistic regression analysis predicting multi-organ distress syndrome.

	Odds Ratio	95% CI	*p* Value
Hemoglobin (g/dL)	0.70553	0.53281–0.93424	0.014
Urine mitochondrial DNA copy number (copies/μL)	1.00022	1.00002–1.00042	0.033

CI, confidence interval.

**Table 4:** AUC, sensitivity, specificity, optimal cut-off values for hemoglobin, urine mitochondrial DNA copy numbers as independent predictors.

	AUC	Sensitivity	Specificity	Optimal Cut-Off Value
Hemoglobin (g/dL)	0.6735	0.48	0.850	10.9500
Urine mitochondrial DNA copy number (copies/μL)	0.7200	0.92	0.525	738.0013

AUC, area under curve.

## Data Availability

The datasets used and/or analyzed in the current study are available from the corresponding author upon reasonable request.

## Abbreviations

The following abbreviations are used in this manuscript:

AKI	Acute kidney injury
Hb	Hemoglobin
MODS	Multi-organ dysfunction syndrome
mtDNA	Mitochondrial DNA
ROS	Reactive oxygen species
DAMP	Damage-associated molecular pattern
TLR9	Toll-like receptor 9
NGAL	Neutrophil gelatinase–associated lipocalin
KIM-1	Kidney injury molecule-1
KDIGO	Kidney disease improving global outcomes
ROC	Receiver operating characteristic
OR	Odds ratio
CI	Confidence interval
